# Pooled prevalence of food away from home (FAFH) and associated non-communicable disease (NCD) markers: a systematic review and meta-analysis

**DOI:** 10.1186/s41043-022-00335-5

**Published:** 2022-11-30

**Authors:** Swapnil Godbharle, Angeline Jeyakumar, Bibek Raj Giri, Hema Kesa

**Affiliations:** 1 Food Evolution Research Laboratory (FERL), School of Tourism and Hospitality Management, College of Business and Economics, University of Johannesburg, Johannesburg, South Africa; 2 Department of Health Sciences, Savitribai Phule Pune University, Ganeshkhind Road, Pune, Maharashtra 411007 India

**Keywords:** Food away from home, Dietary behavior, Non-communicable diseases, Anthropometric changes

## Abstract

**Background:**

Food away from home (FAFH) is an ‘eating behavior’ widely practiced across nations, more common in developed nations. Likewise, in developing countries an increase of close to 50% indicates an upsurge in FAFH consumption. While various indices and tools are used to assess diet quality, diversity, or healthy eating, FAFH is less utilized to study dietary behaviors and the associated disease risk.

**Objective:**

To calculate the pooled estimate of FAFH and identify the associated non-communicable disease (NCD) markers.

**Design:**

Systematic review and meta-analysis.

**Methods:**

Independent electronic searches were conducted across 6 databases: Medline, Web of Science, Scopus, Cochrane library, Ingenta, and CAB direct. Observational studies investigating the association between FAFH and NCD markers published between the year 2011 and 2021 were eligible for inclusion. Studies that included overweight or obese participants, pregnant women, or those under institutional care at baseline were excluded. The guidelines for reporting meta-analysis of observational studies in epidemiology were adhered to in the preparation of this systematic review.

**Results:**

The random effects combined estimate for the overall prevalence of FAFH was 39.96% (95% CI 29.97–53.29). High heterogeneity (*τ*^2^ = 0.63, *I*^2^ = 100%) and high risk of bias were observed among the selected studies. The test for overall effect was observed to be *z* = 25.11 (*p* < 0.001). Eleven out of fourteen studies showed a positive association between FAFH and anthropometric changes. Twelve out of seventeen studies showed a positive association between FAFH and cardiovascular disease (CVD) biomarkers.

**Conclusion:**

Our work confirms FAFH as an evolving dietary behavior in both developing and developed countries, emphasizing the lack of representation from low-income countries. The association of FAFH with obesity and non-communicable disease risk is reinforced by our analyses. These findings should enable policy decisions to meet the rising demand of FAFH with healthier options to prevent the risk of NCD.

**Supplementary Information:**

The online version contains supplementary material available at 10.1186/s41043-022-00335-5.

## Introduction

Food away from home (FAFH) is an ‘eating behavior’ widely practiced across nations, more common in developed nations [1–3]. Likewise, developing countries such as South Africa, India [4, 5], and Mexico [6] report an increase close to 50%, indicating an upsurge in FAFH consumption. In economies under transition, not much difference is observed in FAFH between the rural–urban settings, with the narrowing of the divide. It is well established that eating behavior is influenced by social, physical, and macro-level environments [7]. The distribution of type and number of restaurants in the environment is recognized to affect FAFH. The setting of FAFH is known to influence the energy density of foods and nutrient intake. It is often hypothesized that fast-food outlets provide an obesogenic environment compared to indoor restaurants [8]. Currently, access to food away from home has become easier and has increased with online food delivery [9, 10]. FAFH has been documented to decrease the intake of whole grains, the number of servings of vegetables and milk, and replaced by energy-dense foods with high fat, added sugar, and increased sodium [11–13]. Consequently, FAFH has contributed to dietary acculturation of populations that increase the risk of non-communicable diseases.

The Global Burden of Disease (GBD) endorsed the World Health Organization (WHO) quantification of diet-related non-communicable disease (NCD) risk that one in five deaths could be prevented by addressing the diet-related risk [13]. It further estimated that the disability-adjusted life years (DALY) were highest for smoking, diabetes, and hypertension [14]. Among the outcomes of diet-related risk, high body mass index (BMI), cardiometabolic risk, diabetes, and other NCDs have been widely explored. To demonstrate the causal effect of diet on disease, epidemiological assessments focus on dietary patterns than individual foods or nutrients. Dietary patterns provide a comprehensive representation of the diet and nutrient intake of the population. Although efforts to study the diet-disease association are manifold, evidence is yet to be ascertained. While various indices and tools are used to assess diet quality, diversity, or healthy eating, FAFH is less utilized to study dietary behaviors and the associated disease risk. When studied, it often takes an economic stance such as household income and expenditure on FAFH or is used as a proxy indicator to study poverty in developing countries [15, 16]. Recognizing the need for meta-research to measure specific diet behavior and pattern our work calculated the pooled estimate of FAFH and identified the NCD markers associated with this changing dietary behavior.

## Methods

The guidelines for reporting meta-analysis of observational studies in epidemiology were adhered to in the preparation of this systematic review [17]. Preferred Reporting Items for Systematic Reviews and Meta-Analyses (PRISMA) guidelines were followed [18]. To conduct this review, the published literature was surveyed for observational studies (cohort and cross-sectional) examining the pooled prevalence of consuming food away from home and relationship between eating food away from home and NCD markers.

### Search strategy

Databases searched included Medline, Web of Science, Scopus, Cochrane library, Ingenta, and CAB direct using an advanced search strategy. Literature search was performed between December 2021 and January 2022. The search syntax was elaborated in Medline and adapted to the other databases. A search strategy was developed that used key words indexed by the databases of subject-specific terms related to eating food away from home (e.g., fast foods, convenience or ready-prepared or ready-to-eat or out of home or away from home or outside home or away from home, unhealthy food, fast food or junk food, or restaurant) combined with text words and key words related to non-communicable disease (non-communicable diseases, or non-communicable disorders, non-communicable disease markers or non-communicable disorder markers, hypertension, or high blood pressure, obesity, or high BMI, diabetes).

### Study selection criteria

Observational studies investigating the association between eating food away from home and NCD markers published between the year 2011 and 2021 were eligible for inclusion. To study pooled prevalence, any definition of eating food away from home irrespective of frequency was considered (for example, the definitions using the place of preparation or of consumption of foods) as well as studies, which used a single source of out-of-home foods, e.g., fast foods or school foods. Despite regulation on school food in developed countries, we included these studies as it fit the definition of FAFH. Studies that included terms that did not specify FAFH and used broad terms such as unhealthy foods or convenience foods were reviewed for setting and then selected. To describe the associations between FAFH and NCD biomarkers frequency of FAFH was considered as an exposure. The review targeted free-living humans who were healthy, viz. free from chronic illness and who were not under treatment for ailments at baseline, without specific dietary requirements, from both sexes, from any age, race or ethnicity, and any country. Therefore, studies reporting only on overweight or obese subjects at baseline, pregnant women, or those under institutional care were excluded. Articles reporting on food safety and qualitative papers, such as editorials and comments, were excluded. Studies with a recall period over 12 months and reported anthropometric data were excluded. The references of the articles retained for data extraction were also screened to see whether additional articles emerged (Additional file 1).

### Data extraction

**Fig. 1:**
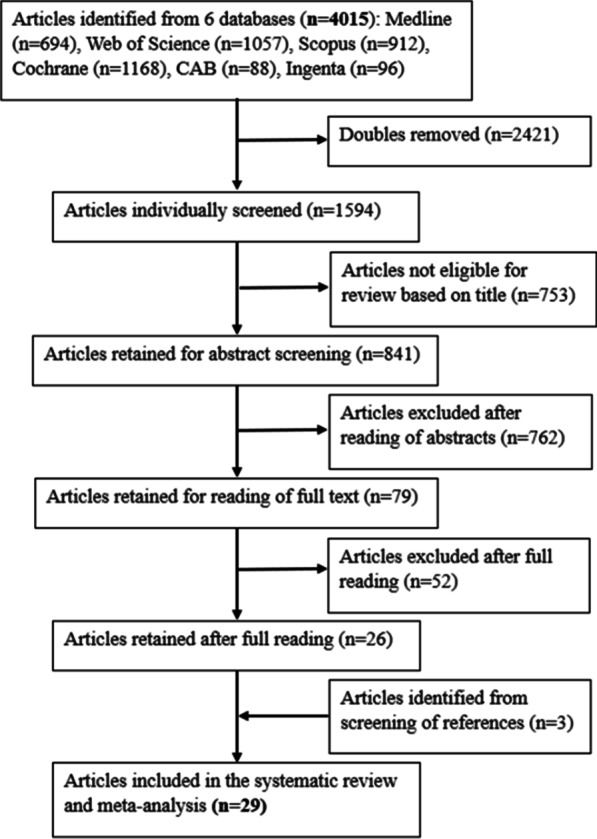
PRISMA chart: Search strategy and selection of studies

All the 4015 articles found were merged into a single database (Mendeley Reference Manager), and duplicates were removed. Two reviewers (SRG and BRG) independently screened each retrieved document for eligibility by examining the titles and the abstracts. In case of doubt at any stage, the paper concerned was kept in the review database. A discussion followed to solve the disagreement between the two independent appraisals, and when necessary, the expertise of a third co-author was requested. A flowchart of the screening is represented in Fig. 1.

Two reviewers (SRG and BRG) independently extracted data using the established eligibility criteria. Information, such as authors and year of publication, countries, participants to the study, and sample size, was extracted to present each study included in this review. Baseline characteristics of the participants, the exposure (frequency of out-of-home eating), and the outcome measures (risk of becoming overweight or obese, BMI, BMI z-score, waist circumference, and other NCD markers) and the main findings were also retrieved. The methodological quality of the studies included in the review was assessed by considering the risk of bias in sample selection (representativeness and participation rate), the design (controlled trial or not), data collection methods (validity and reliability), the appropriateness of statistical tests, and whether they accounted for potential confounders (Additional file 2).

### Methodological quality assessment

Quality rating of systematic reviews and meta-analyses was performed independently by two methodologists using the Quality Assessment Tool for Observational Cohort and Cross-sectional Studies from the National Heart, Lung, and Blood Institute (NHLBI) of the National Institutes of Health [19]. If ratings differed, then reviewers discussed the article in an effort to reach consensus. When consensus was not achieved, the article was forwarded to a third methodologist for adjudication. The Quality Assessment Tool was used to assess each study based on the research question, study population, sample size justification, exposure measurement and timing, outcome measurement, and statistical analyses. The quality assessment tool contained 14 questions in total, so the maximum possible score (MPS) for each study was 14. Every question which received ‘Yes’ as an answer was given a score of one, while every ‘No’ answer was scored zero. Quality of each study was rated as poor (0–4 out of 14 questions), fair (5–10 out of 14 questions), or good (11–14 out of 14 questions) based on the score they received (Additional file 3).

Out of 29 studies, 15 studies scored 11–14 which signified good quality of studies [20–34]. Fourteen studies scored 5–10 which signified fair quality [11, 35–47]. None of the included studies scored 0–4 which signified poor quality.

### Data analysis

Meta-analysis of the selected clinical studies was performed by using random effects model due to the variation between studies [48]. Review Manager (RevMan) software version 5.3 (Cochrane Collaboration, 2014) was used to obtain a forest plot to demonstrate the degree of heterogeneity among the selected studies. The software uses *χ*^2^, *I*^2^, and *τ*^2^ to study heterogeneity among the articles. In this review, reported prevalence in individual articles was extracted, log transformed and standard error of proportion of prevalence was estimated. This model helps in controlling for both unobserved and observed heterogeneity. The log transformation of prevalence from individual studies gives equal weight to the studies. The *p* value is the probability from chi-square statistic calculated using estimates of individual study weight, effect size, and overall effect size. Associations between FAFH and NCD markers from the selected studies were consolidated based on their positive and negative associations and described in our results.

## Results

### Overview of included studies

Of the 4015 articles, 79 were retained for full text reading. Twenty-six articles were kept after full reading and from the screening of their references, three additional studies were retrieved. Hence, 29 papers with a sample, *N* = 437,526, were included in this review (Table 1). All twenty-nine studies had population-based sample and were published in the last 11 years. Of the 29 studies, 15 studies included data from the developed countries, while 14 were from developing countries. Of these, major representations were from China and the USA with 10 and 9 studies, respectively. Other countries were represented with not more than one or two studies.

**Table 1:** Overview of included studies (*N* = 29)

No	Study	Study design	Sampling method	Study location	Sample size (*N*)	Participants	Prevalence of FAFH (%)	FAFH recall period
1	Naska et al. [20]	Prospective cohort study	Random sampling	10 western European countries (Denmark, France, Germany, Greece, Italy, The Netherlands, Norway, Spain, Sweden, and the UK)	24,310 (M-8712; W-15598)	35–74-year-old men and women	42.3	24 h recall for 1 day
2	Anderson et al. [21]	Digit-dialed telephone surveys	Random sampling	USA	4311 (M-1680; W-2631)	18–64-year-old men and women	28	30 days
3	Choi et al. [35]	Empirical study	Stratified systematic cluster sampling	South Korea	1070	Women above 20 years age	30.4	1 month
4	Larson et al. [36]	Observational study	Not mentioned	USA	2287 (M-1030; W-1257)	20–31-year-old men and women	95	7 days
5	Fulkerson et al. [22]	Cross-sectional study	Convenience sampling	USA	1446 (723 adolescents; 723 parents)	11–16-year-old boys and girls	38.4	7 days
6	Smith et al. [37]	Cohort study	Not mentioned	Australia	1896 (M-914; W-982)	26–36-year-old men and women	92.3	7 days
7	Odegaard et al. [11]	Cohort study	Not mentioned	Singapore	43,176	45–74-year-old men and women	40.2	24 h recall for 1 day in past 1 month
8	Buscemi et al. [23]	Observational, Cross-sectional study	Random sampling	Italy	1035	18–90-year-old men and women	85.6	30 days
9	Cahill et al. [24]	Prospective cohort study	Not mentioned	USA	111,631 (M-40789; W-70842)	40–75-year-old men and 30–55-year-old women	54.4	7 days
10	Payab et al. [38]	Empirical study	Cluster sampling	Iran	14,880	6–18-year-old boys’ old girls	25	7 days
11	Bezerra et al. [39]	Empirical study	Random sampling	Brazil	13,736	25–65-year-old men and women	42.7	24 h recall for 2 days
12	Kant et al. [25]	Cross-sectional study	Not mentioned	USA	8314 (M-4070; W-4244)	Men and women above 20 years age	85	7 days
13	Seguin et al. [40]	Cross-sectional study	A stratified random sampling	USA	2001	Men and women above 18 years age	50	7 days
14	Tian et al. [26]	Empirical study	A multistage, random cluster sampling	China	10,633 (M-5084; W-5549)	18–65-year-old men and women	36.5 [30 (in 2004) 43 (in 2011)]	24 h recall for 3 consecutive days
15	Demmler et al. [27]	Cross-section, observational study	Random sampling	Kenya	550	Men and women above 18 years age	53	30 days
16	Dong et al. [28]	Longitudinal cohort study	A multistage, random cluster sampling	China	9454 (3875 children, 2947 mothers, and 2632 fathers)	7–17-year-old children and their parents	42.6	24 h recall for 3 consecutive days (7 days total)
17	Kant and Graubard [29]	Prospective cohort study	Probability sampling	USA	9107	Men and women above 40 years age	90	7 days
18	McClain et al. [41]	A community-based cohort study	A stratified 2-stage probability sample	USA	16,045	18–74-year-old men and women	67.5	24 h recall for 2 consecutive days
19	Zeng and Zeng [43]	Longitudinal cohort study	A multistage, random cluster sampling	China	26,244	18–60-year-old men and women	11 [9.40 (in 2004); 9.89 (in 2006); 11.34 (in 2009); 13.95 (in 2011)]	24 h recall for 3 consecutive days
20	Cunha et al. [42]	Cross-sectional study	Not mentioned	Brazil	5266	10–19-year-old boys and girls	20	24 h recall for 2 consecutive days
21	Choi et al. [30]	Cross-sectional study	Not mentioned	South Korea	640 (M-320; W-320)	20–69-year-old men and women	53.3	24 h recall for 2 days at 3-day interval (weekdays)
22	Liu et al. [44]	Cross-sectional study	Not mentioned	China	8322 (M-3878; W-4444)	Men and women above 18 years age	37.4	24 h recall for 3 consecutive days
23	Wang et al. [31]	Longitudinal cohort study	Not mentioned	China	4518 (M-2441; W-2077)	Men and women above 18 years age	28	24 h recall for 3 days within a week
24	Wang et al. [45]	Cross-sectional study	A multistage, random cluster sampling	China	29,910	18–79-year-old men and women	12.3	7 days
25	Du et al. [32]	Prospective cohort study	A stratified multistage probability sampling	USA	35,084	Men and women above 20 years age	75.9	7 days
26	Chen et al. [46]	Cross-sectional study	Probability random sampling	China	3489	Men and women above 18 years age	19.9 (23.6 in men, and 16.9 in women)	24 h recall on 3 consecutive days
27	Zheng et al. [33]	Cohort study	A stratified cluster sampling	China	3313	7–17-year-old children	80.1	90 days
28	Ma et al. [47]	Cross-sectional study	A multistage stratified sampling	China	15,261 (B-7685; G-7576)	6–17-year-old children	23.2	7 days
29	Cui et al. [34]	Cohort study	A stratified, multistage probability sampling	China	29,597 (M-12088; W-17509)	18–79-year-old men and women	12.4	7 days

Of the total 29 studies, 12 studies each used cross-sectional and cohort study design, while four were empirical study designs that used national data sets. Study participants represented an age range between 6 and 90 years and of both sexes. The sample size at baseline varied from 550 to 1,11,631 participants.

### Quality assessment of the studies

Studies reviewed used varied methods for data collection. The recall period for FAFH ranged from 24 h to 1 year. Twelve studies conducted 24-h dietary recalls. In most studies, anthropometric variables were measured and not self-reported. In almost all the studies, assessment of weight status was done with a standard method or a method which previously established validity and reliability. The studies also used appropriate statistical methods and adjusted for potential confounders, like demographic characteristics and socioeconomic status, non-dietary behavior, such as baseline weight status, energy intake, physical activity, and smoking. Out of the 29 studies reviewed, 5 did not provide any definition of FAFH [11, 23, 30, 35, 36].

### Funnel plot of individual studies selected for meta-analysis

**Fig. 2:**
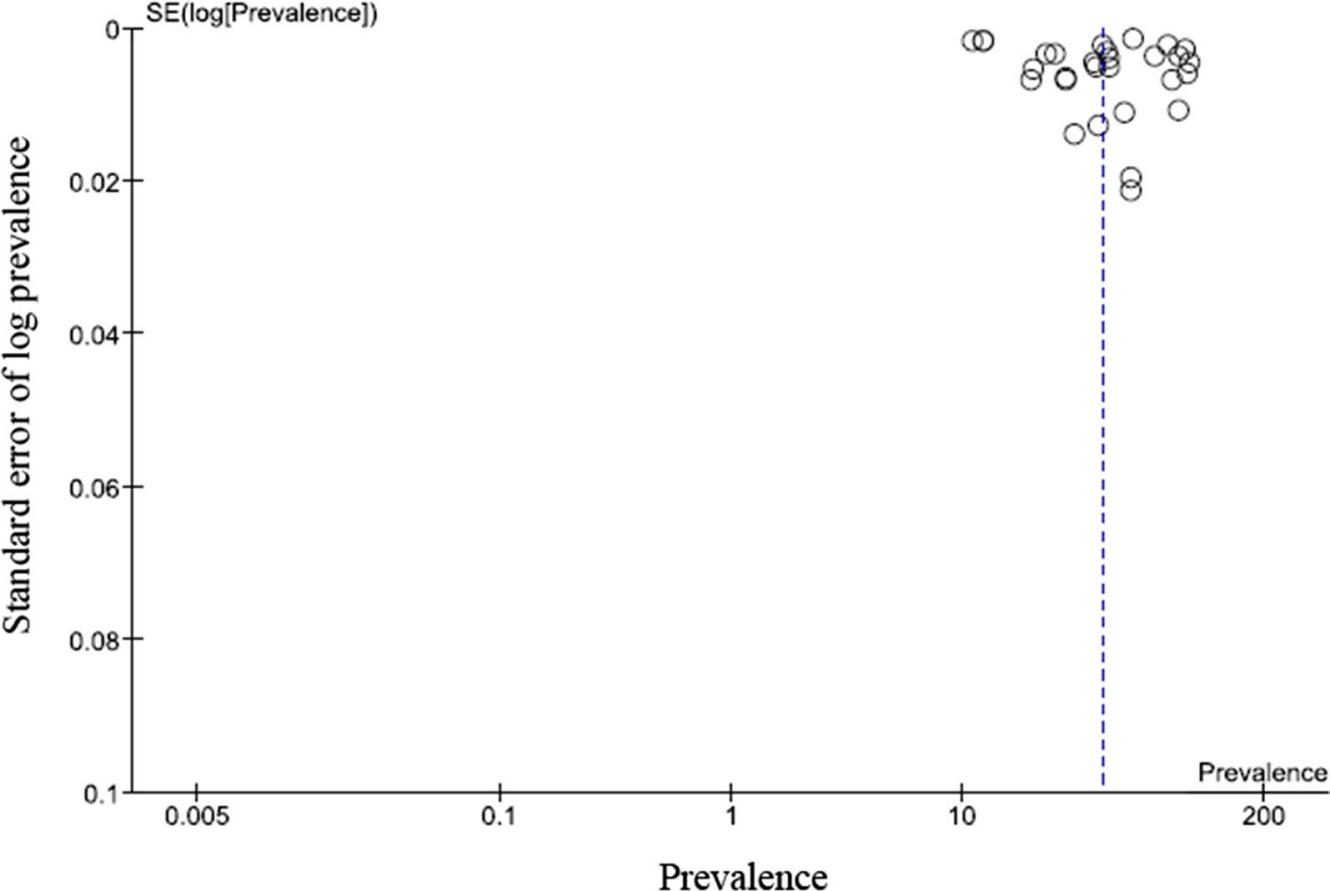
Funnel plot of individual studies selected for meta-analysis. The dotted lines suggest the null hypothesis and the circles represent the individual studies prevalence of FAFH

The asymmetrical distribution of studies in the funnel plot (Fig. 2) provides a visual representation of publication bias.

### Forest plot showing pooled prevalence of food away from home

**Fig. 3:**
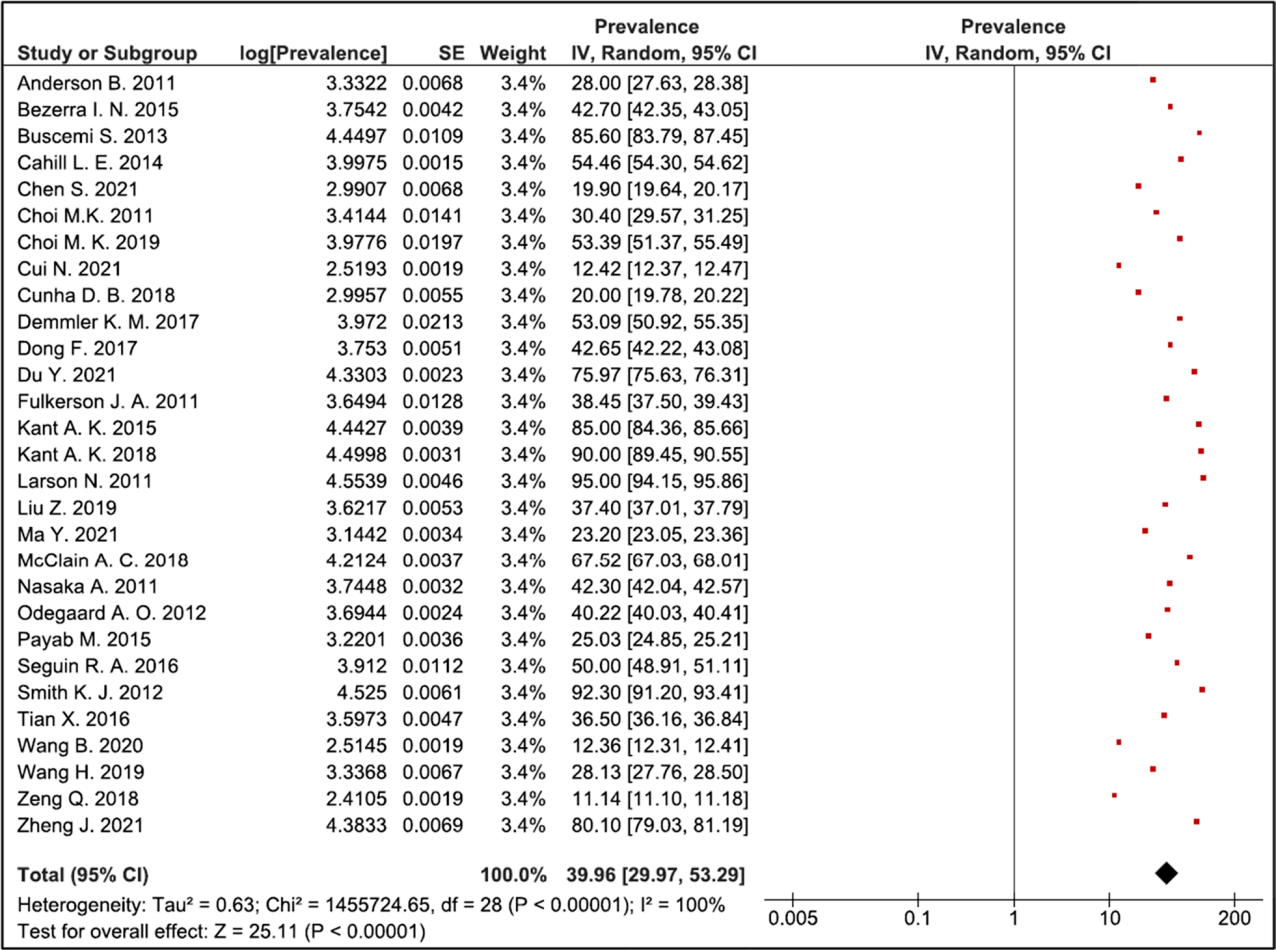
Forest plot showing pooled prevalence of food away from home

Figure 3 shows the forest plot derived for the selected studies. High heterogeneity was observed among the studies (*τ*^2^ = 0.63, *χ*^2^ = 1,455,724.65, *df* = 28, *p* =  < 0.00001, *I*^2^ = 100%). The test for overall effect was observed to be *Z* = 25.11 (*p* < 0.00001). As per categorization of heterogeneity by Higgins et al. 2003, *I*^2^ > 75% indicated considerable heterogeneity. The prevalence of FAFH ranged from 11 to 92% in the reviewed studies. The random effects combined estimate for overall prevalence was 39.96%, (95% CI 29.97–53.29).

### Association of eating food away from home with NCD markers (obesity)

The fourteen articles that studied association between eating food away from home and anthropometric changes assessed FAFH variations in frequency, gender, setting, specific foods, meal occasion or place of FAFH (Table 2). Four studies revealed a positive association between the frequency of eating out and BMI or overweight or obesity [21, 25, 36, 40]. Significant association between meal occasions (*p* < 0.05) specifically lunch and obesity was observed among men [20, 26]. Among the other associations such specific foods, diets, and place of FAFH, identified intake of sweets, foods from on-street vendors, and unhealthy diet patterns even when consumed at home showed significant association with obesity [38, 42]. In contrast to the above observations, two studies [46, 47] did not find any association between eating food away from home and BMI or overweight or obesity. One study [39] found negative association between eating food away from home and obesity. Thus, in our review, eleven out of fourteen studies showed a positive association between FAFH and anthropometric changes.

**Table 2:** Association of eating food away from home with NCD markers (obesity) (*n* = 14)

No	Study	Country	Participants	Baseline characteristics	Exposure	Outcome	Findings
1	Naska et al. [20]	10 western European countries (Denmark, France, Germany, Greece, Italy, The Netherlands, Norway, Spain, Sweden, and the UK)	35–74-year-old men and women	54.45% overall obesity, 39.69% overweight, 14.76% obese	Frequency of eating foods away from home	Change in BMI from baseline to follow-up	Among men, eating at restaurants was significantly associated with BMI and non-significantly with weight gain. Among women no similar patterns were observed
2	Anderson et al. [21]	USA	18–64-year-old men and women	28% ate fast-food ≥ 2 times/week 28.9% overall obesity	Frequency of fast-food consumption	BMI	The prevalence of obesity increased consistently with frequenting fast-food restaurants, from 24% of those going less than once a week to 33% of those going 3 or more times per week
3	Choi et al. [35]	South Korea	Women above 20 years age	30.4% ate away from home 1–6 times a week 31.9% overall obesity	Frequency of eating foods away from home	BMI	Those who ate out were more likely to be obese. A lower obesity rate was observed among housewives with moderate FAFH frequency who had 7–12 years of education, and were younger than 50 years old
4	Larson et al. [36]	USA	20–31-year-old men and women	95% ate from one or more type of restaurant in a given week 51.72% overall obesity, 29.19% overweight, 22.52% obese	Weekly frequency of eating foods away from home	BMI	More frequent use of fast-food restaurants that primarily served burgers and French fries was associated with higher risk for overweight/obesity; higher intake of total energy, sugar-sweetened beverages, and fat, and with lower intake of healthful foods and key nutrients
5	Payab et al. [38]	Iran	6–18-year-old boys’ old girls	9.7% overall obesity, 7% overweight, 12.5% obese	Weekly frequency of junk food consumption	BMI, WC	This study showed significant association between consumption of sweets and both general and abdominal obesity. There was no significant association among junk foods (fast foods and salty snacks) and obesity
6	Bezerra et al. [39]	Brazil	25–65-year-old men and women	27.5% overall obesity, 38% overweight, 17% obese	Frequency of eating foods away from home	BMI	Although AFHF consumption was not related to overweight or obesity status, individuals who consumed foods away from home had higher intakes of energy-dense foods
7	Kant et al. [61]	USA	Men and women above 20 years age	50% of adults reported ⩾ 3 AFH and 35% reported ⩾ 2 fast-food meals/week	Weekly frequency of eating foods away from home	BMI	The mean BMI increased with increasing weekly frequency of AFH meals (*p* = 0.0004); the associations were stronger in ⩾ 50-year-olds relative to < 50-year-olds
8	Seguin et al. [40]	USA	Men and women above 18 years age	16% ate away from home ⩾ 5 time per week	Weekly frequency of eating foods away from home	BMI	Higher frequency of FAFH was associated with higher BMI, after adjusting for age, income, education, race, smoking, marital status, and physical activity (women: 0.001; men: 0.003)
9	Tian et al. [26]	China	18–65-year-old men and women	41.7% overall obesity, 31.7% overweight, 9.9% obese	Weekly frequency of eating foods away from home and restaurant availability	Change in BMI from baseline to follow-up	Higher frequency of eating away from home is positively associated with BMI, but this effect is only significant for men (*p* < 0.05). Moreover, while eating dinner or breakfast away from home contributes to BMI increase for men (*p* < 0.05), no such association is found for lunch
10	McClain et al. [41]	USA	18–74-year-old men and women	47.1% ate away from home ⩾ 5 time per week 76.8% overall obesity, 37.2% overweight, 39.6% obese	Weekly frequency of eating foods away from home	BMI	Study findings identify on-street vendors, but not other types of AFHFs, as being associated with higher odds of obesity
11	Zeng and Zeng [43]	China	18–60-year-old men and women	Not mentioned	Weekly frequency of eating foods away from home	Change in BMI from baseline to follow-up	The results illustrated that the frequency of meals consumed away from home had a significantly positive effect on BMI in urban China, whereas no significant association was observed in rural China
12	Cunha et al. [42]	Brazil	10–19-year-old boys and girls	47.9% ate away from home in a given day. 22.3% overall obesity	Frequency of eating foods away from home	BMI z-score	Only the at-home ‘Western pattern’ was positively associated with BMI z-scores (*β* = 0.0006; < 0.001). Results indicate that unhealthy dietary pattern consumed at home is associated to BMI z-score, while away-from-home food consumption is not associated
13	Zheng et al. [33]	China	7–17-year-old children	80.1% ate away from home ≥ 1 times/week. 29.8% overall obesity	Frequency of eating foods away from home	BMI, WC	Both eating out for Western-style and for Chinese-style food was not statistically significantly associated with overweight risk after adjusting for child and parental factors
14	Ma et al. [47]	China	6–17-year-old children	12.3% ate away from home ≥ 3 times per week 23.8% overall obesity 13.2% overweight, 10.6% obese	Weekly frequency of eating foods away from home	BMI	Findings revealed that eating out three times per week or more was statistically significant associated with higher prevalence of overweight and obesity among boys (OR 1.20, 95 CI 1.04–1.38) compared with those ate out less than three times per week. However, no significantly association was observed among girls (OR 0.91, 95 CI 0.78–1.01)

### Association of eating food away from home with NCD markers (cardio vascular disease biomarkers)

Seventeen out of twenty-nine studies tested association between frequency of FAFH and CVD biomarkers. The biomarkers included all-cause mortality, diabetes, hypertension, carotid atherosclerosis, cholesterol levels, waist–height ratio (WHtR), metabolic syndrome (MetS), and hyperuricemia (Table 3). Of the various biomarkers used in the selected studies, positive association was observed between FAFH and all-cause mortality [11, 32], diabetes [11, 24, 30, 45], CVD biomarkers [23, 27, 28, 37], and hyperuricemia [34, 44, 46]. MetS as another outcome variable [31] showed positive association between the frequency of eating out, specifically in middle-aged males. No association was observed between FAFH and hypertension [22, 23, 27, 37, 38] and CVD biomarkers [25]. Thus, in our review twelve out of seventeen studies showed a positive association between FAFH and CVD biomarkers.

**Table 3:** Association of eating food away from home with NCD markers (CVD biomarkers) (*n* = 17)

No	Study	Country	Participants	Baseline characteristics	Exposure	Outcome	Findings
1	Kant and Graubard [29]	USA	Men and women above 40 years age	33% respondents reported eating ⩾ 3 restaurant prepared meals/week	Weekly frequency of eating foods prepared at restaurants	All-cause and coronary heart disease, cerebrovascular disease and diabetes (cardiometabolic) mortality and cardiometabolic biomarkers	In this study, the risks of mortality from all-causes or cardiometabolic diseases and frequency of eating restaurant prepared meals were unrelated
2	Du et al. [32]	USA	Men and women above 20 years age	3.4% ate away from home ⩾ 2 times a day	Weekly frequency of eating foods away from home	Mortality status (cardiovascular and cancer deaths)	Frequent consumption of meals prepared away from home is significantly associated with increased risk of all-cause mortality. The association of eating meals prepared away from home with cardiovascular mortality and cancer mortality warrants additional investigation
3	Odegaard et al. [11]	Singapore	45–74-year-old men and women	5.6% had presence of diabetes mellitus	Frequency of eating foods away from home	CHD mortality and incident type 2 diabetes mellitus	Western-style fast-food intake is associated with increased risk of developing type 2 diabetes mellitus and of coronary heart disease mortality in an Eastern (Chinese Singaporean) population
4	Wang et al. [45]	China	18–79-year-old men and women	9% had presence of type 2 diabetes	Frequency of eating foods away from home	Type 2 diabetes	An excessive frequency of AFHs was likely to increase the prevalence of T2DM. Meanwhile, BMI partially mediates the effects of the frequency of AFHs on T2DM
5	Cahill et al. [24]	USA	40–75-year-old men and 30–55-year-old women	14.0% and 3.5% of women and 22.6% and 7.4% men reported fried-food consumption 4–6 and ⩾ 7 times/wk., respectively	Frequency of eating foods away from home	Type 2 diabetes and coronary artery disease	Frequent fried-food consumption was significantly associated with risk of incident T2D and CAD (coronary artery disease). These associations were mediated in part by BMI, hypertension, and hypercholesterolemia
6	Choi et al. [30]	South Korea	20–69-year-old men and women	61% ate away from home ⩾ 3 times per week	Frequency of eating foods away from home	BMI, total cholesterol, serum glucose, and insulin	When all confounding factors had been adjusted, the risk of hyperglycemia was significantly lower in participants who rarely dined out compared to participants who dined out one or two times a week. Hence, the frequency of dining out can be related to diabetes risk
7	Buscemi et al. [23]	Italy	18–90-year-old men and women	20% had presence of carotid atherosclerosis	Frequency of eating foods away from home	Carotid intima-media thickness (carotid atherosclerosis), fasting glucose, total cholesterol, HDL cholesterol (HDL-c), triglycerides, uric acid and creatinine concentrations, and blood pressure	Age, gender distribution, BMI and prevalence of hypertension were not significantly different among the three groups, nor was the prevalence of clinically silent carotid atherosclerosis (*p* = 0.85) and the c-IMT (*p* = 0.16). In conclusion, this study shows that saturated fat consumption has no significant impact on carotid atherosclerosis in participants with no history of cardiovascular disease or diabetes
8	Demmler et al. [27]	Kenya	Men and women above 19 years age	15% had presence of pre-diabetes	Frequency of eating foods away from home	BMI, blood pressure, and fasting blood glucose	This study suggests that buying food in supermarkets increases BMI, fasting blood glucose, and the probability of being overweight/obese, pre-diabetic, and suffering from the metabolic syndrome
9	Payab et al. [38]	Iran	6–18-year-old boys and girls	9.75% overall obesity, 7% overweight, 12.5% obese	Weekly frequency of junk food consumption	BMI, WC, and blood pressure	This study showed significant association between consumption of sweets and both general and abdominal obesity. There was no significant association between sweets consumption, sweetened beverages intake, junk foods (fast foods and salty snacks) and hypertension
10	Fulkerson et al. [22]	USA	11–16-year-old adolescents and their parents	65% ate away from home 3 to 6 times per week 25.6% Overweight/Obese among adolescents and 56.3% among parents	Weekly frequency of eating foods away from home	Chronic Disease biomarkers (percent body fat; cholesterol, HDL cholesterol, LDL cholesterol, triglycerides, glucose, insulin, and blood pressure)	Study findings indicate that the odds of overweight/obesity are considerably greater when families report at least one weekly away-from-home dinner purchase. Mean percent body fat and CVD biomarkers (Mean percent body fat, metabolic risk cluster z-scores, and insulin levels) are also considerably greater with weekly purchases of family dinner from fast-food restaurants and takeout sources
11	Smith et al. [37]	Australia	26–36-year-old men and women	39.1% of men and 20.0% of women consumed takeaway food twice a week or more	Weekly frequency of takeaway food consumption	Cardiometabolic risk factors (blood pressure, triglycerides, total cholesterol, HDL cholesterol, LDL cholesterol, and glucose)	Consuming takeaway food at least twice a week was associated with cardiometabolic risk factors in women but less so, in men. The effect of takeaway food consumption was attenuated when adjusted for obesity
12	Kant et al. [25]	USA	Men and women above 20 years age	50% of adults reported ⩾ 3 AFH and 35% reported ⩾ 2 fast-food meals/week	Weekly frequency of eating foods away from home	Metabolic biomarkers (BMI, serum cholesterol, triglycerides, glycohemoglobin, and fasting glucose	Reporters of frequent AFH and fast-food meals had higher BMI and lower concentrations of HDL cholesterol; however, profiles of other biomarkers did not indicate higher metabolic risk. However, the serum concentrations of nutrients with mostly plant foods as sources declined with increasing AFH meal frequency
13	Dong et al. [28]	China	7–17-year-old children and their parents	43% ate away from home ⩾ 1 times per day	Frequency of eating foods away from home	Cardiometabolic disease (CMD) risk factors (blood pressure, glycated hemoglobin, and C-reactive protein)	Away-from-home eating was related to a higher WHtR in children but a lower WHtR in parents, likely due to different food choices and responses to urbanization between 2 generations in China
14	Wang et al. [31]	China	Men and women above 18 years age	18% ate away from home > 0 to ⩾ 3 times in last 3 days	Frequency of eating foods away from home	BMI, WC, blood pressure, serum HDL cholesterol, serum TGs levels, and fasting plasma glucose	Middle-aged males were prone to get MetS when eating out frequently, while young females were more likely to reduce their risk of getting MetS when eating out very often. In particular, EAFH was associated with a lower risk of getting high serum triglycerides (TGs), abdominal adiposity, elevated blood pressure, and impaired fasting blood glucose for young females, while higher risk of high serum TGs, abdominal adiposity, elevated blood pressure, and impaired fasting blood glucose for middle-aged males (all * p* < 0.05)
15	Liu et al. [44]	China	Men and women above 18 years age	18% ate away from home ⩾ 1 times in a given day The prevalence of HU is 15.4% in the total population (11.0% for female and 20.4% for male)	Frequency of eating foods away from home	Hyperuricemia (high serum uric acid levels)	In this study, we found that EAFH is associated with HU in China. After adjusting confounding factors and sensitivity analysis, the correlation still exists. Stratified by age, gender, and BMI, we further found one more important result: obesity (BMI ≥ 24), male, and middle-aged people who eat out are at higher risks of HU. In conclusion, EAFH is positively associated with the prevalence of HU
16	Chen et al. [46]	China	Men and women above 18 years age	The frequency of EAFH was 19.9%. The proportion of high serum uric acid was 26.4% in the total sample, 32.5% in men, and 21.2% in women	Frequency of eating foods away from home	Hyperuricemia (high serum uric acid levels)	The current study suggested a 1.27-fold OR of high serum uric acid in adults who had a habit of EAFH, compared with those without EAFH. A positive association was found in men, but not in women. Adults eating out during breakfast or at a restaurant were inclined to be associated with an increased OR of high serum uric acid. This study found that EAFH was associated with an increased odds ratio of high serum uric acid in men, but not in women
17	Cui et al. [34]	China	18–79-year-old men and women	The frequency of FAFH was 12%. The proportion of high serum uric acid was 12% in the total sample	Frequency of eating foods away from home	Hyperuricemia (high serum uric acid levels)	Our findings indicated that eating out was associated with increased SUA levels and elevated hyperuricemia risk in rural China, especially in males. Moreover, the relationship was partly mediated by BMI

## Discussion

### Outcome measures

#### Pooled prevalence

Food consumed outside home has emerged as a dietary pattern that has drawn the collective attention of public health researchers and policymakers. Our meta-analysis identified a pooled estimate of nearly 40% of the population frequently consuming FAFH which emphasizes the rising concern. Among the selected 29 studies the prevalence ranged from 11 to 92%. As per the World Bank’s categorization, of developed and developing nations our screening identified an equal representation of studies from both economies and no representation from low-income countries. This connotes the significance of economics in such dietary behaviors. This further unfolds the variations in the purpose of studies. While developed nations focused on consumer utilization of FAFH and the associated expenditure, developing countries often used expenditure surveys on FAFH to define poverty. FAFH has thus arrived as an interdisciplinary indicator and offers more scope in epidemiological measurements.

The studies selected for the meta-analysis assessed diverse outcomes. These include: all-cause mortality, anthropometric changes such as BMI and waist circumference, and clinical parameters such as hypertension and specific markers in lipid profile were few. Further, indicators of blood glucose such as pre-diabetes, random blood sugar, and hyperuricemia (an indicator of metabolic syndrome, diabetes mellitus, CVD, and chronic renal failure) [49] were used as NCD markers in two studies.

#### All-cause mortality

In our review, all-cause mortality as an outcome of this dietary pattern showed a positive association in one of the studies and a negative association in another [29, 32]. This contrasting evidence arises from a developed country (the USA) with a prospective cohort as the study design. A systematic review that investigated the association between diet patterns and all-cause mortality concluded that nutrient-dense diets decreased the risk of all-cause mortality. Given that this meta-analysis used data from 28 nations with high human development indices (HDI), the conclusions are highly specific  [50]. If greater percent of the household expenditure is utilized for FAFH, quality of foods consumed could be compromised [51]. These findings raise serious concern as the socioeconomic gradient in obesity is attributable to the setting or food environment that affects the poorer sections of the populations and identifies the need to study the effect of dietary behavior in such settings [52, 53]. Work by Du and co-workers explained the combined effect of FAFH along with race, ethnicity, and low family income with all-cause mortality.

#### Anthropometric changes

BMI was used as an indicator in fourteen of the studies in combination with biomarkers. Positive association between the frequency of consumption of FAFH and increased BMI was observed in six studies. However, three studies showed no association or negative association between FAFH and BMI. Of these, two were performed among a selective population of either children or women [43, 54]. Although earlier work among children have identified no association, the change in dietary pattern among school children is already a public health concern [55, 56]. The study that showed a negative association selected for the analysis was from Korea, where the prevalence of obesity among men and women was far below the limits to achieve the Millennium Development Goals (MDGs) [57]. Lack of association between FAFH and BMI has been reported in earlier studies as well [58, 59]. Definition of FAFH if broad is likely to affect the findings. Evidence from a systematic review on FAFH and anthropometric changes specifically fast-food restaurants and restaurant foods positively predicted BMI increase among women [60]. Irrespective of developed or developing country status, Kenya [27], the USA [24, 61, 62], and China [44, 45] showed significant associations with FAFH and high BMI. Similar associations have been established by studies from different regions included in this analysis. In these studies, the association between economic growth and food behavior manifested in anthropometrics and other biomarkers but not with hypertension [27, 38]. FAFH being a time-trend data, longitudinal studies are likely to demonstrate strong associations.

#### NCD risk

Contradictory findings emerged between gender and cardiovascular risk. Males were at a higher risk of impaired fasting glucose and hyperuricemia (as an indicator of metabolic syndrome and NCD risk) [34, 46] in two of the selected studies and young females were at less risk of metabolic syndrome [45], and these results aligned with published literature [63, 64]. Such differences were observed between BMI and FAFH. The WHO explains that such differences could be attributed to the risk associated behavior such as tobacco smoking, and alcohol intake combined with the changing dietary patterns [65].

Articles selected in our review showed a significant association between types of meals, the timing of food, and the FAFH setting such as a grocery store, restaurant, or street food, and NCD risk [11, 24, 27, 41]. It also emphasized that unhealthy food even when consumed at home increases the risk [42]. While BMI and fast foods are widely studied [66, 67], few studies have explored eating out and cardiometabolic risk, especially specific diet patterns such as fast- or fried-food consumption. Likewise, dietary constituents such as amount and type of fat are widely studied [68]. Variations in the associations are likely to be observed as dietary measurements in every study varied to assess FAFH. Recall bias, factor analysis, and consolidation of food items into groups are few possibilities for such variations. Specifically, with eating out behavior, increased frequency of FAFH emerged as a contributing factor in majority of the studies. The results emphasize the negative association between suboptimal diets consumed out of home on NCD biomarkers. However, in our review FAFH along with other factors contributed to the increased risk of NCD. Among the demographic factors, level of education [30, 40], race, sedentary behavior [40], age, and male gender [31, 44] were contributing factors. Other mediating factors were BMI as reported in seven studies [27, 34, 38, 44, 45, 61, 62]. Western dietary pattern and urbanization are indicated in few studies, with time constraints for food preparation [11, 28, 33, 42].

### Heterogeneity in FAFH definitions

Our review identified interchangeable terms such as AFHF, AFH, and EAFH. Certain definitions included cooked meals that covered the meals away from home. It is projected that proxy indicators for FAFH such as expenditure for food away from home are likely to underestimate the dietary behavior, as the measurement is often restricted to one question. The heterogeneity in the intra-household meal patterns highlights the scope of measurement error likely to occur in single measurements [5]. Definitions ranged from describing the frequency of fast-food consumption [21, 35], to specific meals such as breakfast or dinner, irrespective of the type of service [22]. Few analyses defined eating at work as a separate category as this could be the food carried from home or food vendors. And eating at home included eating on special occasions [20]. Despite the heterogeneity in definitions, the tool used to capture FAFH in any of the above terms was the 24-h diet recall. The usage of this tool nevertheless varied with the study designs. It varied from a single 24-h recall in cross-sectional studies to multiple recalls over 3 months. While cross-sectional studies poorly captured the association between NCD risk and FAFH, longitudinal studies and cross-sectional analysis of national data yielded better associations [32, 45].

Our review indicated that irrespective of the status of development, an increase in economic activities, long working hours, increased mobility, and a higher percentage of women choosing careers contribute to FAFH and its associated expenditure. The inelastic characteristics of expenditure on FAFH with the transition, combined with the poor control over informal vendors, add to the nutritional concern in developing countries [4]. Thus, there are more reasons to analyze FAFH and health outcomes at the backdrop of risk transition.

## Strengths and limitations

The strength of this analyses is the large sample, covering developed and developing countries and the combination of anthropometric, cardiovascular, and diabetes markers as outcomes that had a higher predictive potential. Our work is not free from limitations. As the focus of our investigation was to estimate FAFH behavior, the selection of studies was limited to two common criteria, FAFH and its frequency. Further, pooled estimate from individual studies is likely to under estimate the risk behavior. Restrictions were not applied to the type of foods consumed, setting, and the nutrient intake such as energy or fat and its association with NCD risk, as this would have restricted the number of studies selected. Similarly, restrictions were not specified for age groups or outcomes that resulted in a high degree of heterogeneity. Thus, it is likely that study with younger age groups had children included in the sample that could have contributed to the broad confidence intervals in the pooled estimate. Few studies without clear study design or a calculated a sample size that were included could distort the findings substantially. Our selection did not include studies from low-income countries and therefore limits the generalization of our findings. The funnel plot shows asymmetry where the concentration of studies indicates significant bias and therefore was not subjected to further statistical tests as it could lead to misleading inferences [69]. Selective publication of positive results, longer time needed to publish negative results, and exclusion of other language manuscripts are few biases that could have affected our study results [70]. Our work was restricted to study the prevalence of FAFH, and FAFH as risk of NCD and CVD is described as secondary outcomes. Five studies [32–34, 46, 47] included in our analysis were published in 2021. Access to FAFH during the COVID-19 pandemic was significantly affected, and this could have affected our findings and interpretations. This review was not registered in any of the systematic review registries that is likely to introduce bias. However, all the necessary guidelines for reporting meta-analysis of observational studies in epidemiology were adhered to in the preparation of this analysis.

### Implications for research and practice

In summary, our work confirms FAFH as an evolving dietary behavior in both developing and developed countries, emphasizing the lack of representation from low-income countries. The association of FAFH with obesity and non-communicable disease risk is reinforced by our analyses. These findings should enable policy decisions to meet the rising demand of FAFH with healthier options to prevent the risk of NCD. The multi-disciplinary use of FAFH offers much scope to identify diet behavior and disease in epidemiological studies. There is a critical need to generate evidence using longitudinal studies, between FAFH and other dietary exposures in unregulated settings in low- and middle-income countries with mortality outcomes.

## Supplementary Information

**Additional file 1**. PRISMA checklist.

**Additional file 2**. Search strategy.

**Additional file 3**. Quality assessment of the included studies.

## Data Availability

All data generated or analyzed during this study are included in this published article.

## Abbreviations

FAFH: Food away from home
GBD: Global Burden of Disease
WHO: World Health Organization
NCD: Non-communicable disease
DALY: Disability-adjusted life years
BMI: Body Mass Index
PRISMA: Preferred Reporting Items for Systematic Reviews and Meta-Analyses
NHLBI: National Heart, Lung, and Blood Institute
MPS: Maximum possible score
CVD: Cardiovascular disease
WHtR: Waist–height ratio
MetS: Metabolic syndrome
CHD: Coronary heart disease
HDL: High-density lipoprotein
HDI: Human Development Index
MDGs: Millennium development goals
