# Supplementary material for: Pooled prevalence of food away from home (FAFH) and associated non-communicable disease (NCD) markers: a systematic review and meta-analysis
Source: J Health Popul Nutr. 2022 Nov 30;41:55. doi: 10.1186/s41043-022-00335-5 (PMC9709732; doi:10.1186/s41043-022-00335-5)
Supplement: Supplementary file 2 — Additional file 2. Search strategy. [file 41043_2022_335_MOESM2_ESM.docx]

Search number, Query, Sort By, Filters, Search Details, Results, Time

25,(out of home food) AND (high BMI),,,"""out""[All Fields] AND (""home environment""[MeSH Terms] OR (""home""[All Fields] AND ""environment""[All Fields]) OR ""home environment""[All Fields] OR ""home""[All Fields]) AND (""food""[MeSH Terms] OR ""food""[All Fields]) AND (""high""[All Fields] AND ""BMI""[All Fields])",32,04:07:24

24,(out of home food) AND (obesity),,,"""out""[All Fields] AND (""home environment""[MeSH Terms] OR (""home""[All Fields] AND ""environment""[All Fields]) OR ""home environment""[All Fields] OR ""home""[All Fields]) AND (""food""[MeSH Terms] OR ""food""[All Fields]) AND (""obeses""[All Fields] OR ""obesity""[MeSH Terms] OR ""obesity""[All Fields] OR ""obese""[All Fields] OR ""obesities""[All Fields] OR ""obesity s""[All Fields])",232,04:07:01

23,(out of home food) AND (diabetes),,,"""out""[All Fields] AND (""home environment""[MeSH Terms] OR (""home""[All Fields] AND ""environment""[All Fields]) OR ""home environment""[All Fields] OR ""home""[All Fields]) AND (""food""[MeSH Terms] OR ""food""[All Fields]) AND (""diabete""[All Fields] OR ""diabetes mellitus""[MeSH Terms] OR (""diabetes""[All Fields] AND ""mellitus""[All Fields]) OR ""diabetes mellitus""[All Fields] OR ""diabetes""[All Fields] OR ""diabetes insipidus""[MeSH Terms] OR (""diabetes""[All Fields] AND ""insipidus""[All Fields]) OR ""diabetes insipidus""[All Fields] OR ""diabetic""[All Fields] OR ""diabetics""[All Fields] OR ""diabets""[All Fields])",75,04:06:39

22,(out of home food) AND (high blood pressure),,,"""out""[All Fields] AND (""home environment""[MeSH Terms] OR (""home""[All Fields] AND ""environment""[All Fields]) OR ""home environment""[All Fields] OR ""home""[All Fields]) AND (""food""[MeSH Terms] OR ""food""[All Fields]) AND (""hypertension""[MeSH Terms] OR ""hypertension""[All Fields] OR (""high""[All Fields] AND ""blood""[All Fields] AND ""pressure""[All Fields]) OR ""high blood pressure""[All Fields])",30,04:06:18

21,(out of home food) AND (hypertension),,,"""out""[All Fields] AND (""home environment""[MeSH Terms] OR (""home""[All Fields] AND ""environment""[All Fields]) OR ""home environment""[All Fields] OR ""home""[All Fields]) AND (""food""[MeSH Terms] OR ""food""[All Fields]) AND (""hypertense""[All Fields] OR ""hypertension""[MeSH Terms] OR ""hypertension""[All Fields] OR ""hypertension s""[All Fields] OR ""hypertensions""[All Fields] OR ""hypertensive""[All Fields] OR ""hypertensive s""[All Fields] OR ""hypertensives""[All Fields])",26,04:05:48

20,(food away from home) AND (high BMI),,,"(""food""[MeSH Terms] OR ""food""[All Fields]) AND ""away""[All Fields] AND (""home environment""[MeSH Terms] OR (""home""[All Fields] AND ""environment""[All Fields]) OR ""home environment""[All Fields] OR ""home""[All Fields]) AND (""high""[All Fields] AND ""BMI""[All Fields])",20,04:05:26

19,(food away from home) AND (obesity),,,"(""food""[MeSH Terms] OR ""food""[All Fields]) AND ""away""[All Fields] AND (""home environment""[MeSH Terms] OR (""home""[All Fields] AND ""environment""[All Fields]) OR ""home environment""[All Fields] OR ""home""[All Fields]) AND (""obeses""[All Fields] OR ""obesity""[MeSH Terms] OR ""obesity""[All Fields] OR ""obese""[All Fields] OR ""obesities""[All Fields] OR ""obesity s""[All Fields])",153,04:05:01

18,(food away from home) AND (diabetes),,,"(""food""[MeSH Terms] OR ""food""[All Fields]) AND ""away""[All Fields] AND (""home environment""[MeSH Terms] OR (""home""[All Fields] AND ""environment""[All Fields]) OR ""home environment""[All Fields] OR ""home""[All Fields]) AND (""diabete""[All Fields] OR ""diabetes mellitus""[MeSH Terms] OR (""diabetes""[All Fields] AND ""mellitus""[All Fields]) OR ""diabetes mellitus""[All Fields] OR ""diabetes""[All Fields] OR ""diabetes insipidus""[MeSH Terms] OR (""diabetes""[All Fields] AND ""insipidus""[All Fields]) OR ""diabetes insipidus""[All Fields] OR ""diabetic""[All Fields] OR ""diabetics""[All Fields] OR ""diabets""[All Fields])",23,04:04:37

17,(food away from home) AND (high blood pressure),,,"(""food""[MeSH Terms] OR ""food""[All Fields]) AND ""away""[All Fields] AND (""home environment""[MeSH Terms] OR (""home""[All Fields] AND ""environment""[All Fields]) OR ""home environment""[All Fields] OR ""home""[All Fields]) AND (""hypertension""[MeSH Terms] OR ""hypertension""[All Fields] OR (""high""[All Fields] AND ""blood""[All Fields] AND ""pressure""[All Fields]) OR ""high blood pressure""[All Fields])",18,04:04:10

16,(food away from home) AND (hypertension),,,"(""food""[MeSH Terms] OR ""food""[All Fields]) AND ""away""[All Fields] AND (""home environment""[MeSH Terms] OR (""home""[All Fields] AND ""environment""[All Fields]) OR ""home environment""[All Fields] OR ""home""[All Fields]) AND (""hypertense""[All Fields] OR ""hypertension""[MeSH Terms] OR ""hypertension""[All Fields] OR ""hypertension s""[All Fields] OR ""hypertensions""[All Fields] OR ""hypertensive""[All Fields] OR ""hypertensive s""[All Fields] OR ""hypertensives""[All Fields])",13,04:03:45

15,(fast food consumption) AND (non-communicable disorder markers),,,"(""fast foods""[MeSH Terms] OR (""fast""[All Fields] AND ""foods""[All Fields]) OR ""fast foods""[All Fields] OR (""fast""[All Fields] AND ""food""[All Fields]) OR ""fast food""[All Fields]) AND (""consumptions""[All Fields] OR ""economics""[MeSH Terms] OR ""economics""[All Fields] OR ""consumption""[All Fields]) AND (""non-communicable""[All Fields] AND (""disease""[MeSH Terms] OR ""disease""[All Fields] OR ""disorder""[All Fields] OR ""disorders""[All Fields] OR ""disorder s""[All Fields] OR ""disordes""[All Fields]) AND (""biomarkers""[MeSH Terms] OR ""biomarkers""[All Fields] OR ""marker""[All Fields] OR ""markers""[All Fields]))",3,04:03:23

14,(fast food consumption) AND (non-communicable disease markers),,,"(""fast foods""[MeSH Terms] OR (""fast""[All Fields] AND ""foods""[All Fields]) OR ""fast foods""[All Fields] OR (""fast""[All Fields] AND ""food""[All Fields]) OR ""fast food""[All Fields]) AND (""consumptions""[All Fields] OR ""economics""[MeSH Terms] OR ""economics""[All Fields] OR ""consumption""[All Fields]) AND ((""noncommunicable diseases""[MeSH Terms] OR (""noncommunicable""[All Fields] AND ""diseases""[All Fields]) OR ""noncommunicable diseases""[All Fields] OR (""non""[All Fields] AND ""communicable""[All Fields] AND ""disease""[All Fields]) OR ""non communicable disease""[All Fields]) AND (""biomarkers""[MeSH Terms] OR ""biomarkers""[All Fields] OR ""marker""[All Fields] OR ""markers""[All Fields]))",5,04:02:43

13,(fast food consumption) AND (non-communicable disorders),,,"(""fast foods""[MeSH Terms] OR (""fast""[All Fields] AND ""foods""[All Fields]) OR ""fast foods""[All Fields] OR (""fast""[All Fields] AND ""food""[All Fields]) OR ""fast food""[All Fields]) AND (""consumptions""[All Fields] OR ""economics""[MeSH Terms] OR ""economics""[All Fields] OR ""consumption""[All Fields]) AND (""non-communicable""[All Fields] AND (""disease""[MeSH Terms] OR ""disease""[All Fields] OR ""disorder""[All Fields] OR ""disorders""[All Fields] OR ""disorder s""[All Fields] OR ""disordes""[All Fields]))",57,04:02:10

12,(fast food consumption) AND (non-communicable diseases),,,"(""fast foods""[MeSH Terms] OR (""fast""[All Fields] AND ""foods""[All Fields]) OR ""fast foods""[All Fields] OR (""fast""[All Fields] AND ""food""[All Fields]) OR ""fast food""[All Fields]) AND (""consumptions""[All Fields] OR ""economics""[MeSH Terms] OR ""economics""[All Fields] OR ""consumption""[All Fields]) AND (""noncommunicable diseases""[MeSH Terms] OR (""noncommunicable""[All Fields] AND ""diseases""[All Fields]) OR ""noncommunicable diseases""[All Fields] OR (""non""[All Fields] AND ""communicable""[All Fields] AND ""diseases""[All Fields]) OR ""non communicable diseases""[All Fields])",123,04:01:43

11,(out of home food) AND (non-communicable disorder markers),,,"""out""[All Fields] AND (""home environment""[MeSH Terms] OR (""home""[All Fields] AND ""environment""[All Fields]) OR ""home environment""[All Fields] OR ""home""[All Fields]) AND (""food""[MeSH Terms] OR ""food""[All Fields]) AND (""non-communicable""[All Fields] AND (""disease""[MeSH Terms] OR ""disease""[All Fields] OR ""disorder""[All Fields] OR ""disorders""[All Fields] OR ""disorder s""[All Fields] OR ""disordes""[All Fields]) AND (""biomarkers""[MeSH Terms] OR ""biomarkers""[All Fields] OR ""marker""[All Fields] OR ""markers""[All Fields]))",0,04:01:20

10,(out of home food) AND (non-communicable disorder markers) - Schema: all,,,"""out""[All Fields] AND ""home""[All Fields] AND ""food""[All Fields] AND (""non-communicable""[All Fields] AND ""disorder""[All Fields] AND ""markers""[All Fields])",0,04:01:20

9,(out of home food) AND (non-communicable disease markers),,,"""out""[All Fields] AND (""home environment""[MeSH Terms] OR (""home""[All Fields] AND ""environment""[All Fields]) OR ""home environment""[All Fields] OR ""home""[All Fields]) AND (""food""[MeSH Terms] OR ""food""[All Fields]) AND ((""noncommunicable diseases""[MeSH Terms] OR (""noncommunicable""[All Fields] AND ""diseases""[All Fields]) OR ""noncommunicable diseases""[All Fields] OR (""non""[All Fields] AND ""communicable""[All Fields] AND ""disease""[All Fields]) OR ""non communicable disease""[All Fields]) AND (""biomarkers""[MeSH Terms] OR ""biomarkers""[All Fields] OR ""marker""[All Fields] OR ""markers""[All Fields]))",0,04:00:19

8,(out of home food) AND (non-communicable disease markers) - Schema: all,,,"""out""[All Fields] AND ""home""[All Fields] AND ""food""[All Fields] AND (""non-communicable""[All Fields] AND ""disease""[All Fields] AND ""markers""[All Fields])",0,04:00:19

7,(out of home food) AND (non-communicable disorders),,,"""out""[All Fields] AND (""home environment""[MeSH Terms] OR (""home""[All Fields] AND ""environment""[All Fields]) OR ""home environment""[All Fields] OR ""home""[All Fields]) AND (""food""[MeSH Terms] OR ""food""[All Fields]) AND (""non-communicable""[All Fields] AND (""disease""[MeSH Terms] OR ""disease""[All Fields] OR ""disorder""[All Fields] OR ""disorders""[All Fields] OR ""disorder s""[All Fields] OR ""disordes""[All Fields]))",9,03:59:56

6,(out of home food) AND (non-communicable diseases),,,"""out""[All Fields] AND (""home environment""[MeSH Terms] OR (""home""[All Fields] AND ""environment""[All Fields]) OR ""home environment""[All Fields] OR ""home""[All Fields]) AND (""food""[MeSH Terms] OR ""food""[All Fields]) AND (""noncommunicable diseases""[MeSH Terms] OR (""noncommunicable""[All Fields] AND ""diseases""[All Fields]) OR ""noncommunicable diseases""[All Fields] OR (""non""[All Fields] AND ""communicable""[All Fields] AND ""diseases""[All Fields]) OR ""non communicable diseases""[All Fields])",19,03:59:17

5,(food away from home) AND (non-communicable disorder markers),,,"(""food""[MeSH Terms] OR ""food""[All Fields]) AND ""away""[All Fields] AND (""home environment""[MeSH Terms] OR (""home""[All Fields] AND ""environment""[All Fields]) OR ""home environment""[All Fields] OR ""home""[All Fields]) AND (""non-communicable""[All Fields] AND (""disease""[MeSH Terms] OR ""disease""[All Fields] OR ""disorder""[All Fields] OR ""disorders""[All Fields] OR ""disorder s""[All Fields] OR ""disordes""[All Fields]) AND (""biomarkers""[MeSH Terms] OR ""biomarkers""[All Fields] OR ""marker""[All Fields] OR ""markers""[All Fields]))",0,03:58:40

4,(food away from home) AND (non-communicable disorder markers) - Schema: all,,,"""food""[All Fields] AND ""away""[All Fields] AND ""home""[All Fields] AND (""non-communicable""[All Fields] AND ""disorder""[All Fields] AND ""markers""[All Fields])",0,03:58:40

3,(food away from home) AND (non-communicable disease markers),,,"(""food""[MeSH Terms] OR ""food""[All Fields]) AND ""away""[All Fields] AND (""home environment""[MeSH Terms] OR (""home""[All Fields] AND ""environment""[All Fields]) OR ""home environment""[All Fields] OR ""home""[All Fields]) AND ((""noncommunicable diseases""[MeSH Terms] OR (""noncommunicable""[All Fields] AND ""diseases""[All Fields]) OR ""noncommunicable diseases""[All Fields] OR (""non""[All Fields] AND ""communicable""[All Fields] AND ""disease""[All Fields]) OR ""non communicable disease""[All Fields]) AND (""biomarkers""[MeSH Terms] OR ""biomarkers""[All Fields] OR ""marker""[All Fields] OR ""markers""[All Fields]))",1,03:58:04

2,(food away from home) AND (non-communicable disorders),,,"(""food""[MeSH Terms] OR ""food""[All Fields]) AND ""away""[All Fields] AND (""home environment""[MeSH Terms] OR (""home""[All Fields] AND ""environment""[All Fields]) OR ""home environment""[All Fields] OR ""home""[All Fields]) AND (""non-communicable""[All Fields] AND (""disease""[MeSH Terms] OR ""disease""[All Fields] OR ""disorder""[All Fields] OR ""disorders""[All Fields] OR ""disorder s""[All Fields] OR ""disordes""[All Fields]))",6,03:57:02

1,(food away from home) AND (non-communicable diseases),,,"(""food""[MeSH Terms] OR ""food""[All Fields]) AND ""away""[All Fields] AND (""home environment""[MeSH Terms] OR (""home""[All Fields] AND ""environment""[All Fields]) OR ""home environment""[All Fields] OR ""home""[All Fields]) AND (""noncommunicable diseases""[MeSH Terms] OR (""noncommunicable""[All Fields] AND ""diseases""[All Fields]) OR ""noncommunicable diseases""[All Fields] OR (""non""[All Fields] AND ""communicable""[All Fields] AND ""diseases""[All Fields]) OR ""non communicable diseases""[All Fields])",10,03:56:21
