# Supplementary material for: Pooled prevalence of food away from home (FAFH) and associated non-communicable disease (NCD) markers: a systematic review and meta-analysis
Source: J Health Popul Nutr. 2022 Nov 30;41:55. doi: 10.1186/s41043-022-00335-5 (PMC9709732; doi:10.1186/s41043-022-00335-5)
Supplement: Supplementary file 3 — Additional file 3. Quality assessment of the included studies. [file 41043_2022_335_MOESM3_ESM.docx]

**Table 2: Quality assessment of the studies included in the review**

| **Study** | **Question number** | | | | | | | | | | | | | | **Total score** | **Study quality** |
| --- | --- | --- | --- | --- | --- | --- | --- | --- | --- | --- | --- | --- | --- | --- | --- | --- |
|  | **1** | **2** | **3** | **4** | **5** | **6** | **7** | **8** | **9** | **10** | **11** | **12** | **13** | **14** |  |  |
| Nasaka A. et al., 2011 | Y | Y | Y | Y | N | Y | Y | Y | Y | N | Y | N | Y | Y | 11 | Good |
| Anderson B. et al., 2011 | Y | Y | Y | Y | N | Y | Y | Y | Y | N | Y | N | Y | Y | 11 | Good |
| Choi M.K. et al., 2011 | Y | Y | Y | Y | N | N | Y | Y | Y | N | Y | N | NR | Y | 9 | Fair |
| Larson N. et al., 2011 | Y | Y | Y | Y | N | Y | Y | N | Y | N | Y | N | Y | Y | 10 | Fair |
| Fulkerson J. A. et al., 2011 | Y | Y | Y | Y | Y | Y | N | Y | Y | Y | Y | N | Y | Y | 12 | Good |
| Smith K. J. et al., 2012 | Y | Y | Y | Y | N | N | N | Y | N | N | Y | N | Y | Y | 8 | Fair |
| Odegaard A. O. et al., 2012 | Y | Y | Y | Y | N | N | Y | N | Y | N | Y | N | Y | Y | 9 | Fair |
| Buscemi S. et al., 2013 | Y | Y | Y | Y | Y | N | Y | Y | Y | Y | Y | N | Y | Y | 12 | Good |
| Cahill L. E. et al., 2014 | Y | Y | Y | Y | Y | Y | N | Y | Y | N | Y | N | Y | Y | 11 | Good |
| Payab M. et al., 2015 | Y | Y | Y | Y | N | N | Y | Y | Y | N | Y | N | Y | Y | 10 | Fair |
| Bezerra I. N. et al., 2015 | Y | Y | Y | Y | N | Y | Y | N | N | N | Y | N | NR | N | 7 | Fair |
| Kant A. K. et al., 2015 | Y | Y | Y | Y | Y | Y | N | Y | Y | Y | Y | N | Y | Y | 12 | Good |
| Seguin R. A. et al., 2016 | Y | Y | Y | Y | N | N | N | N | Y | N | Y | N | Y | Y | 8 | Fair |
| Tian X. et al., 2016 | Y | Y | Y | Y | N | Y | Y | Y | N | Y | Y | N | Y | Y | 11 | Good |
| Demmler K. M. et al., 2017 | Y | Y | Y | Y | N | Y | Y | Y | Y | Y | Y | N | Y | Y | 12 | Good |
| Dong F. et al., 2017 | Y | Y | Y | Y | Y | Y | Y | N | Y | Y | Y | N | Y | Y | 12 | Good |
| Kant A. K. et al., 2018 | Y | Y | Y | Y | Y | N | N | Y | Y | Y | Y | N | Y | Y | 11 | Good |
| McClain A. C. et al., 2018 | Y | Y | Y | Y | N | N | Y | N | N | N | Y | N | Y | N | 7 | Fair |
| Zeng Q. et al., 2018 | Y | Y | Y | Y | N | Y | Y | Y | Y | N | Y | N | NR | Y | 10 | Fair |
| Cunha D. B. et al., 2018 | Y | Y | Y | Y | N | Y | N | Y | Y | N | Y | N | Y | Y | 10 | Fair |
| Choi M. K. et al., 2019 | Y | Y | Y | Y | Y | Y | N | Y | Y | N | Y | N | Y | Y | 11 | Good |
| Liu Z. et al., 2019 | Y | Y | Y | Y | Y | N | Y | N | Y | Y | Y | N | Y | N | 10 | Fair |
| Wang H. et al., 2019 | Y | Y | Y | Y | N | Y | Y | Y | Y | Y | Y | N | Y | Y | 12 | Good |
| Wang B. et al., 2020 | Y | Y | Y | Y | N | Y | Y | N | Y | N | Y | N | Y | Y | 10 | Fair |
| Du Y. et al., 2021 | Y | Y | Y | Y | Y | Y | Y | Y | Y | Y | Y | N | Y | Y | 13 | Good |
| Chen S. et al., 2021 | Y | Y | Y | Y | N | N | N | Y | N | N | Y | N | Y | Y | 8 | Fair |
| Zheng J. et al., 2021 | Y | Y | Y | Y | N | Y | Y | Y | Y | N | Y | N | Y | Y | 11 | Good |
| Ma Y. et al., 2021 | Y | Y | Y | Y | Y | Y | N | N | Y | N | Y | N | Y | Y | 10 | Fair |
| Cui N. et al., 2021 | Y | Y | Y | Y | N | Y | Y | Y | Y | Y | Y | N | Y | Y | 12 | Good |
| **Questions for quality assessment**   1. Was the research question or objective in this paper clearly stated? 2. Was the study population clearly specified and defined? 3. Was the participation rate of eligible persons at least 50%? 4. Were all the subjects selected or recruited from the same or similar populations? 5. Was a sample size justification, power description, or variance and effect estimates provided? 6. For the analyses in this paper, were the exposure(s) of interest measured prior to the outcome(s) being measured? 7. Was the timeframe sufficient so that one could reasonably expect to see an association between exposure and outcome if it existed? 8. For exposures that can vary in amount or level, did the study examine different levels of the exposure? 9. Were the exposure measures (independent variables) clearly defined, valid, reliable, and implemented consistently across all study participants? 10. Was the exposure(s) assessed more than once over time? 11. Were the outcome measures (dependent variables) clearly defined, valid, reliable, and implemented? consistently across all study participants? 12. Were the outcome assessors blinded to the exposure status of participants? 13. Was loss to follow-up after baseline 20% or less? 14. Were key potential confounding variables measured and adjusted statistically for their impact on the relationship? between exposure(s) and outcome(s)? | | | | | | | | | | | | | | | | |

**Quality was rated as poor (0–4 out of 14 questions), fair (5–10 out of 14 questions), or good (11–14 out of 14 questions); Y: yes, N: no, NA: not applicable, NR: not reported**
